# Inhibin-Mediated Regulation of Folliculogenesis and Litter Size in Small Ruminants and Non-Ruminants

**DOI:** 10.3390/biology15201831

**Published:** 2026-10-13

**Authors:** Abd Ullah, Chunming Wang, Ying Han, Guiling Cao, Muhammad Zahoor Khan, Changfa Wang

**Affiliations:** College of Agriculture and Biology, Liaocheng University, Liaocheng 252000, China

**Keywords:** inhibin, litter size, reproductive efficiency, prolificacy, genetic associations, livestock

## Abstract

Litter size is a critical determinant of reproductive efficiency and economic productivity in livestock breeding. Inhibin, a glycoprotein hormone of the transforming growth factor-β (TGF-β) superfamily, plays a pivotal role in regulating follicle-stimulating hormone (FSH) secretion through negative feedback within the hypothalamic–pituitary–gonadal axis. By modulating FSH levels, inhibin influences follicular recruitment, maturation, and ovulation rate—key processes that determine litter size. This review provides a comprehensive analysis of inhibin isoforms (*inhibin A* and *inhibin B*), their antagonistic relationship with activin signaling, and their species-specific roles in reproductive regulation. Genetic studies in small ruminants and non-ruminants indicate that polymorphisms in inhibin-related genes, particularly *INHA*, may contribute to variation in prolificacy-related traits; however, the strength and consistency of these associations differ among species and populations. This review further integrates findings from functional validation studies, particularly in mouse models, demonstrating that partial reduction of inhibin bioactivity can enhance FSH secretion, promote follicular recruitment, and increase ovulation rate and litter size, whereas excessive disruption of inhibin signaling may impair reproductive efficiency. Importantly, the biological effects of inhibin modulation depend on the degree of pathway alteration and the balance between follicular recruitment and prenatal survival. Although inhibin represents an important endocrine regulator of reproductive performance, current genomic evidence suggests that litter-size variation is controlled by multiple interacting pathways, and the contribution of inhibin-related genes requires further functional validation. Future integration of genomic, endocrine, and functional approaches will be essential to clarify the role of inhibin pathways and determine their potential application in sustainable livestock breeding strategies.

## Simple Summary

In livestock farming, the number of healthy young animals raised in each pregnancy shapes both the food we produce and the livelihoods of the people who keep these animals. Rather than reporting new experiments, this review gathers and compares what is already known about one of the body’s own fertility signals—a hormone made in the ovaries that helps to determine how many eggs a female releases at a time—across the animals that farmers depend on most, such as goats, sheep, and pigs, alongside lessons learned from laboratory mice. We aim to turn this scattered evidence into a clearer, more usable picture for breeders and researchers. In particular, we explain why simply pushing for the largest possible litters can backfire, because very large litters may harm the health of both mothers and their newborns, and why a more balanced approach tends to have better and more sustainable results. We also highlight where current understanding is weakest—most clearly in rabbits—so that future work can be directed where it is most needed. Through this synthesis, we aim to support breeding decisions that improve farm productivity while centering animal health and welfare.

## 1. Introduction

Reproductive traits are of primary interest in livestock because they directly influence the biological and economic efficiency of animal production systems [1,2,3,4]. Follicle-stimulating hormone (FSH) and luteinizing hormone (LH) play central roles in mammalian reproduction by regulating follicular growth, ovulation, and gametogenesis [1,5]. A critical aspect of reproductive regulation is hormonal control, in which FSH and LH drive ovarian synthesis of estradiol and progesterone, thereby preparing the reproductive tract for fertilization, implantation, and pregnancy [6,7]. In contrast, inhibin primarily functions as a negative regulator of pituitary FSH synthesis and secretion [8,9]. This negative feedback mechanism is crucial for regulating the number of antral follicles recruited into the growing follicular cohort from which ovulatory follicles are selected, and thereby the ovulation rate, which directly affects litter size in both ruminant and non-ruminant species [5,8,9,10]. Understanding inhibin’s role in reproductive health is therefore crucial for improving reproductive outcomes in livestock.

**Figure 1:**
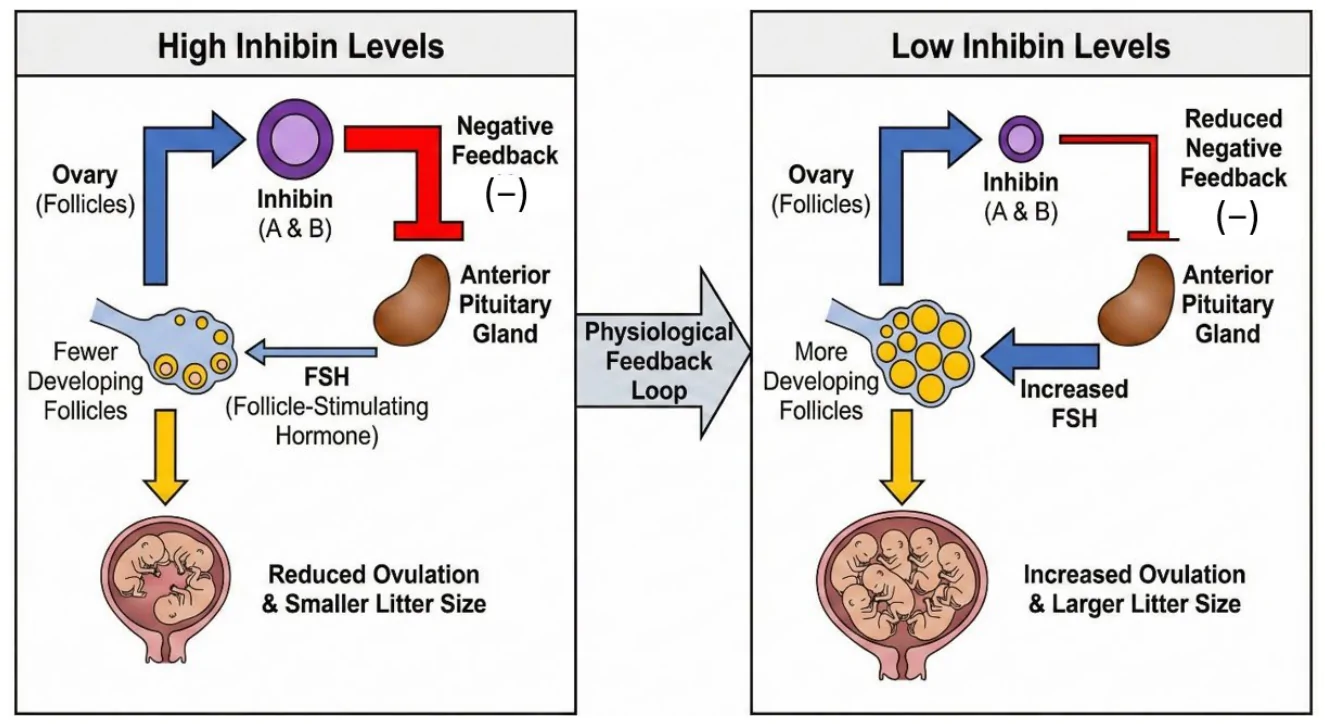
Conceptual relationship between inhibin levels, pituitary follicle-stimulating hormone (FSH) secretion, follicular recruitment, and potential litter size. Blue arrows indicate stimulatory or directional pathways, red T-shaped lines indicate negative feedback inhibition, and yellow arrows indicate progression toward ovulation and litter-size outcomes. Higher inhibin levels enhance negative feedback on pituitary FSH and restrict follicular recruitment, whereas reduced inhibin activity permits increased FSH secretion and recruitment of additional follicles [39]. Increased ovulation may increase potential litter size, although the final number of viable offspring also depends on subsequent reproductive survival [11].

One strategy to enhance female reproductive efficiency in multiparous species is to increase litter size [11]. Inhibin is produced in the ovary, principally by granulosa cells of growing antral follicles, with its synthesis being particularly influenced by feedback regulation within the hypothalamic–pituitary–ovarian axis [12,13]. Inhibin secretion helps maintain balanced FSH levels, which in turn support proper ovarian follicle development and maturation [8,14,15]. The contrasting physiological consequences of higher versus lower inhibin activity for pituitary FSH secretion, follicular recruitment, ovulation rate, and potential litter size are illustrated in Figure 1.

In ruminants such as cattle, sheep, and goats, and in non-ruminant species such as pigs, inhibin’s contribution to follicular recruitment and ovulation rate has been documented [16,17,18,19]. This review examines the underlying mechanisms of inhibin’s action, its impact on ovulation rate and litter size, and its potential as a target for enhancing litter size in livestock. Litter size, defined as the number of offspring produced per birthing event, is a complex quantitative trait shaped by a diverse array of genes involved in reproductive and developmental pathways, as well as by sequential physiological events including ovulation, fertilization, embryo development, and fetal survival [20,21,22]. It is therefore a crucial economic trait, because the number of offspring produced per pregnancy directly determines output per breeding female and farm profitability [20,21]. Litter size and ovulation rate can be influenced by specific genes with large phenotypic effects, collectively termed fecundity (Fec) genes [23,24]. These include FecB, caused by a missense mutation in *BMPR1B* (Q249R) that alters BMP receptor signaling and increases ovulation rate in Booroola Merino sheep; FecX mutations affecting the oocyte-derived growth factor *BMP15*; FecG mutations involving *GDF9*; and FecL, which is associated with ectopic ovarian expression of *B4GALNT2* [25,26,27,28,29]. These major fecundity genes regulate follicular development through distinct mechanisms within the TGF-β superfamily pathway, including changes in granulosa-cell differentiation, follicular responsiveness to intra-ovarian signals, and oocyte–somatic cell communication, rather than through direct regulation of inhibin synthesis or secretion [25,26,27,28]. However, the *FecL* locus represents an important exception, as ectopic *B4GALNT2* expression in granulosa cells alters inhibin A glycosylation and has been shown to reduce its bioactivity, demonstrating an interaction between fecundity pathways and inhibin signaling [29].

Understanding the hormonal regulation of follicular recruitment and ovulation is therefore directly relevant to breeding strategies aimed at improving prolificacy [30,31]. Inhibin modulates pituitary FSH secretion and thereby influences follicular dynamics and ovulation rate [18,19,32]; however, reproductive outcomes can vary with the extent of inhibin neutralization, and an increased ovulation rate does not necessarily translate into improved fertility [32,33,34,35,36,37,38]. The present review therefore focuses on the physiological role of endogenous inhibin in follicular dynamics, ovulation rate, and litter size across ruminant and non-ruminant species.

## 2. Molecular Structure and Function of Inhibin in Reproduction

### 2.1. Molecular Identity and Isoforms of Inhibin

**Figure 2:**
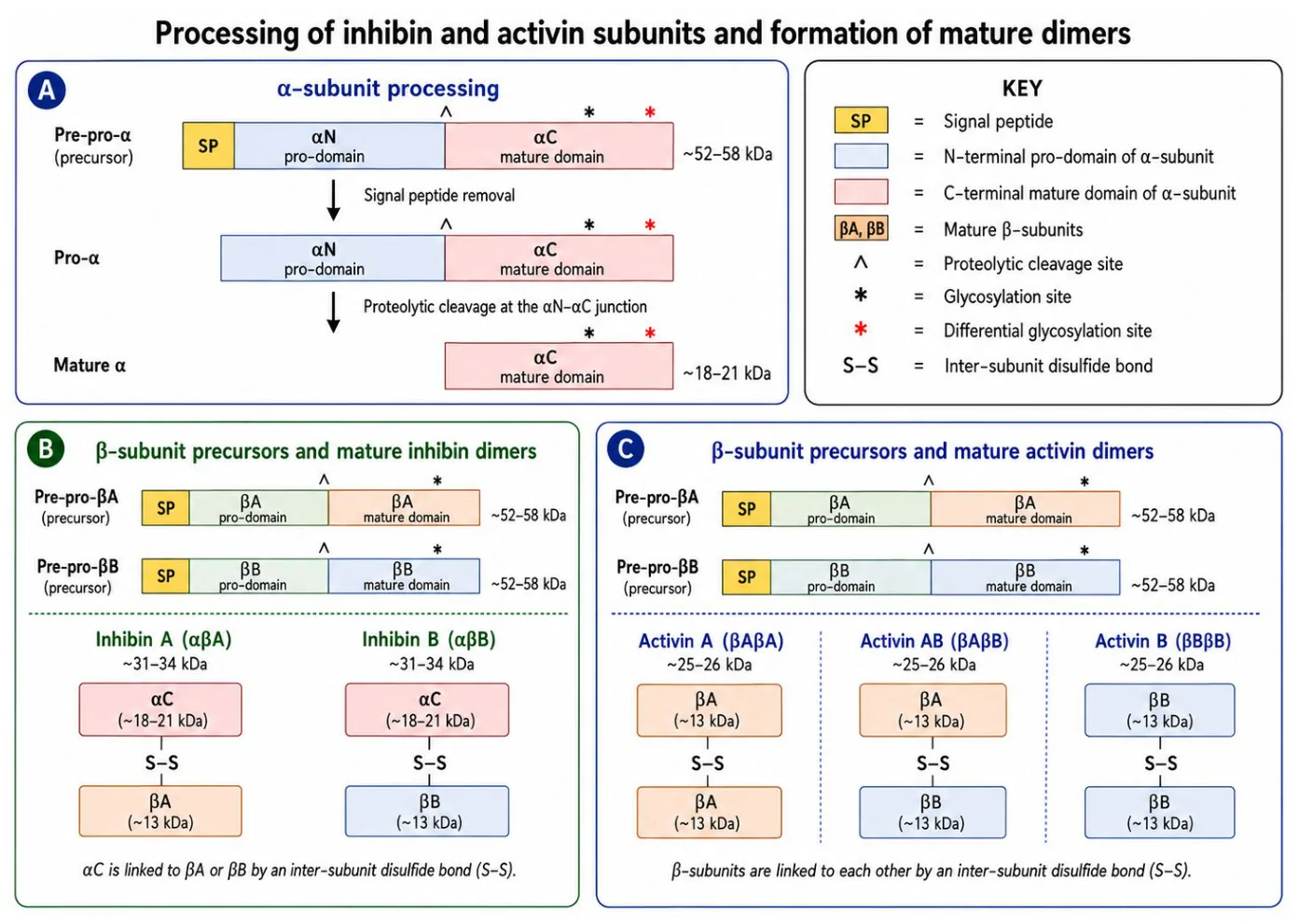
Processing of inhibin and activin subunits and formation of mature dimers. (**A**) Schematic processing of the inhibin α-subunit precursor. Following removal of the signal peptide (SP), the α-subunit precursor undergoes proteolytic processing to generate the C-terminal mature αC domain. The α-subunit contains N-linked glycosylation sites, including a constitutively glycosylated site and a differentially glycosylated site that contributes to the molecular-mass heterogeneity of mature inhibins [47]. (**B**) The βA- and βB-subunits are synthesized as precursor proteins containing an N-terminal prodomain and a C-terminal mature β-domain separated by a proprotein-convertase cleavage site. Mature inhibin A is formed by covalent association of αC with βA, whereas mature inhibin B consists of αC linked to βB through an inter-subunit disulfide bond. Mature inhibin A and B occur predominantly as approximately 31–34-kDa forms, depending principally on differential glycosylation of the α-subunit [47]. For clarity, glycosylation symbols are shown at the subunit-processing level and are not repeated on the assembled mature dimers. N-linked glycosylation of the inhibin α-subunit contributes to the molecular heterogeneity and secretion of mature inhibin A, whereas glycosylation has a different role in activin A assembly and secretion [47,48]. (**C**) Proteolytic processing of βA and βB precursors generates mature β-subunits, which assemble as βAβA, βAβB, or βBβB dimers to form activin A, activin AB, and activin B, respectively [49,50]. The schematic was redrawn and adapted by the authors based on Makanji et al. [39].

Inhibin is a glycoprotein hormone that is central to the control of reproduction across species. In 1932, McCullagh postulated the existence of a gonadal factor that suppresses FSH secretion by the anterior pituitary; however, inhibin itself was not purified until the mid-1980s [39,40]. The overall precursor-to-dimer scheme is summarized in Figure 2. At the molecular level, inhibins belong to the transforming growth factor-β (TGF-β) superfamily and are mature heterodimeric glycoproteins composed of a common α-subunit linked by a disulfide bond to either a βA- or a βB-subunit [41,42,43]. The mature inhibin α-subunit comprises 134 amino acid residues, while the mature βA- and βB-subunits contain 116 and 115 residues, respectively [44,45]; these lengths provide context for interpreting the residue positions and variants summarized in Table 1 and Table 2. The α- and β-subunits are commonly reported to have approximate molecular masses of 20 kDa and the β-subunits approximately 13 kDa (as shown in Figure 2); however, mature circulating inhibin is generally described as approximately 32 kDa because its molecular mass may vary depending on proteolytic processing and glycosylation [39,46]. Based on β-subunit composition, inhibin occurs in two major isoforms: inhibin A, composed of αβA, and inhibin B, composed of αβB [36]. The same two β-subunits also dimerize with each other to form the activins (βAβA, βAβB, βBβB); inhibin and activin therefore share subunits but exert opposing effects on pituitary FSH, with inhibin suppressing and activin stimulating FSH biosynthesis [46]. Their receptor-level mechanisms are discussed further in Section 2.4. The precursor-processing steps and mature dimer combinations of the α-, βA-, and βB-subunits are shown in Figure 2.

Each mature inhibin subunit contains the characteristic TGF-β-family cystine-knot domain, in which conserved cysteine residues form intrachain disulfide bonds that stabilize the mature subunit structure [39,41,42,43]. In mature inhibin, the α-subunit and βA- or βB-subunit are additionally joined by an inter-subunit disulfide bond [39,41,42,43].

### 2.2. Genes Encoding the Inhibin Subunits: Genomic Organization, Transcriptional Control, and Post-Transcriptional Regulation

Separate genes encode the three inhibin/activin subunits: *INHA* encodes the α-subunit, whereas *INHBA* and *INHBB* encode the βA- and βB-subunits, respectively [39,40]. Each subunit is synthesized as a larger pre–pro-protein comprising an N-terminal signal peptide, a pro-domain, and a C-terminal mature domain; proteolytic cleavage at conserved dibasic (RXXR) sites releases the mature peptides, which assemble into the disulfide-linked heterodimers inhibin A (αβA) and inhibin B (αβB), as depicted in Figure 2 [39,41,42]. The genes are compact, with the mature-peptide coding sequence concentrated in the terminal exon, and orthologues of all three subunits are present across the ruminant and non-ruminant species considered here [39,40]. Because the βA- and βB-subunits are shared between inhibins and activins, sequence variation in *INHBA* or *INHBB* can in principle alter both inhibin and activin dimers simultaneously—an important consideration when interpreting the reproductive effects of β-subunit polymorphisms (Section 3).

Transcription of the inhibin subunit genes is hormonally and developmentally regulated during follicular maturation [51,52]. In granulosa cells, FSH signaling through the Gs-coupled receptor increases intracellular cAMP and activates downstream pathways, including phosphorylation of CREB, which contributes to the transcriptional regulation of *INHA* [53,54]. The *INHA* promoter integrates gonadotropin-responsive signals through transcription factors such as SF-1/NR5A1 and CREB, which have been demonstrated to directly regulate α-subunit promoter activity [55,56,57]. GATA4 also functions as an important ovarian transcription factor involved in granulosa-cell gene regulation and regulation of reproductive gene networks [58,59]. Furthermore, FOXL2 and SMAD-dependent signaling pathways activated by TGF-β superfamily ligands contribute to granulosa-cell differentiation and modulate inhibin/activin-related gene expression, although their direct regulation of the *INHA* promoter remains less clearly established [60,61,62]. The β-subunit genes are also regulated by gonadotropin-dependent and local ovarian signals. FSH/cAMP signaling increases *INHBA* expression and promoter activity in granulosa cells [56,57]. *INHBB* transcription initiates from alternative upstream and downstream transcription start sites separated by approximately 1.05 kb, generating distinct 4.3-kb and 3.3-kb mRNAs; FSH and cAMP-elevating agents preferentially increase transcription of the 4.3-kb transcript relative to the 3.3-kb transcript [63]. Both transcripts encode the same βB-chain and differ in the length of their 5′-untranslated regions [63]; therefore, alternative transcription start sites are currently considered to generate transcript variants that primarily differ in their 5′-UTR structure without altering the encoded βB protein sequence. Local TGF-β-family signals can further modulate β-subunit expression in granulosa cells [57,64].

Timing of transcription: Expression is stage-dependent and underlies differential secretion of the two isoforms. βB/inhibin B predominates in the cohort of small growing follicles, whereas βA/inhibin A rises in the selected dominant follicle and corpus luteum [41,65]. Beyond transcription, the abundance of inhibin subunit transcripts is shaped by post-transcriptional mechanisms, although these remain poorly characterized in livestock. In ovine granulosa cells, miR-134-3p targets *INHBA* and modulates proliferation and apoptosis [34]. Quantitative data on inhibin subunit mRNA half-lives, 3′-UTR-mediated stability, and alternative polyadenylation of *INHA*, *INHBA* and *INHBB* transcripts are largely absent, and this gap limits mechanistic interpretation of the non-coding variants summarized in Section 3.

### 2.3. Inhibin Within the Hypothalamic–Pituitary–Ovarian Axis

**Figure 3:**
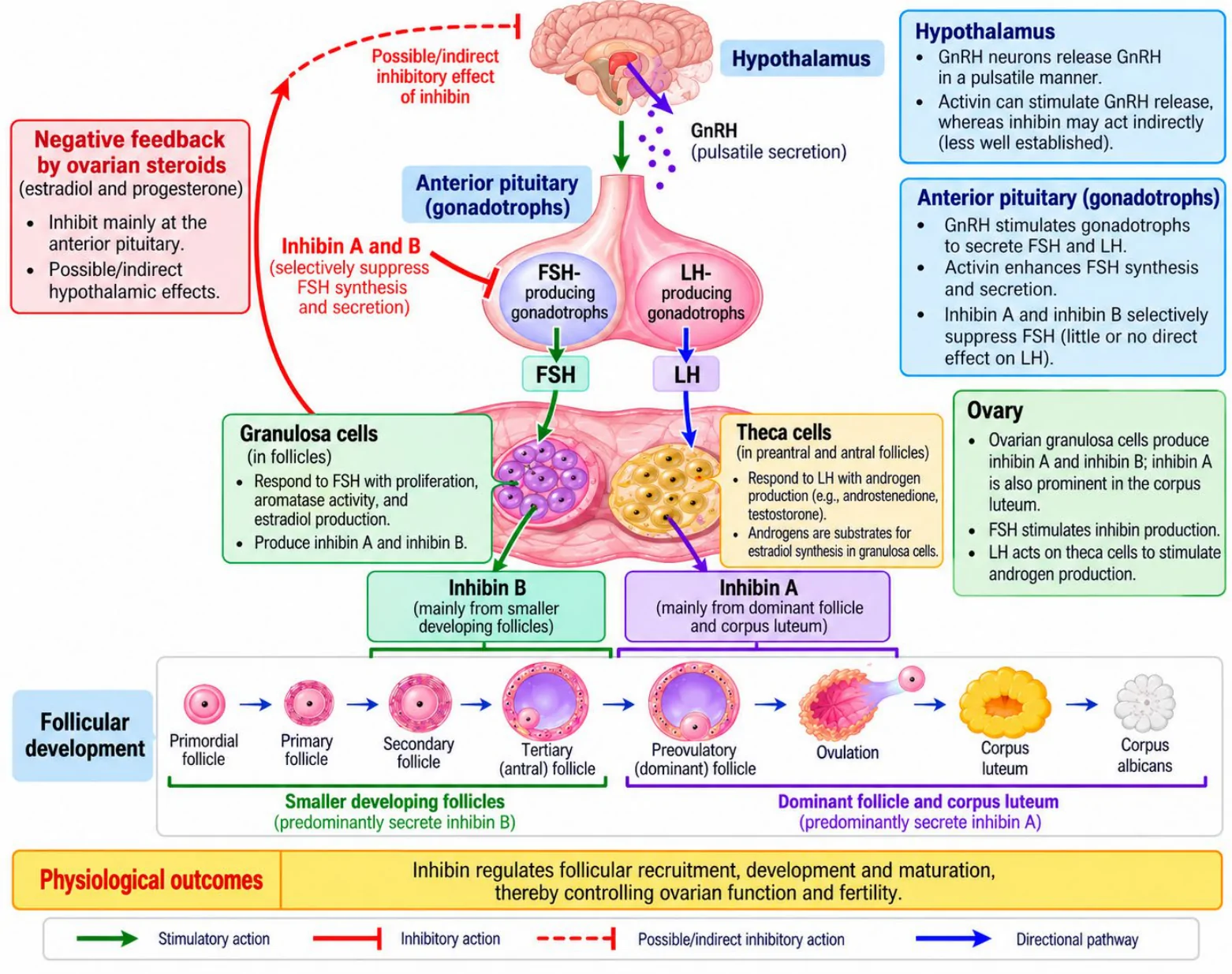
Inhibin regulation of follicular development within the hypothalamic–pituitary–ovarian axis. Gonadotropin-releasing hormone (GnRH) released from the hypothalamus stimulates the anterior pituitary to secrete follicle-stimulating hormone (FSH) and luteinizing hormone (LH). FSH acts primarily on granulosa cells to support follicular growth and development, whereas LH acts mainly on theca cells and contributes to steroidogenesis, ovulation, and luteal function. Inhibin B is produced predominantly by granulosa cells of smaller growing follicles, whereas inhibin A becomes more prominent in the dominant follicle and corpus luteum [66,83,84]. Inhibin A and inhibin B selectively suppress pituitary FSH synthesis and secretion, thereby regulating follicular recruitment and development [66,67]. In addition, inhibin may indirectly influence hypothalamic GnRH regulation through antagonism of activin signaling; this pathway is considered a possible/indirect feedback mechanism [67]. The lower part of the figure illustrates sequential follicular development, ovulation, corpus luteum formation, and luteal regression, highlighting the stage-dependent predominance of inhibin B and inhibin A. Reduced inhibin-mediated feedback decreases suppression of pituitary FSH and may consequently enhance follicular recruitment and development. The schematic was created by the authors using BioRender.com based on physiological relationships summarized from the cited literature.

As illustrated in Figure 3, inhibin–activin regulation of FSH operates within the hypothalamic–pituitary–ovarian (HPO) axis. Inhibin is a glycoprotein hormone synthesized predominantly by ovarian granulosa cells (and, to a lesser extent, theca-associated follicular compartments), and it serves as a key regulator of mammalian reproductive function [46]. Functionally, inhibin A and inhibin B act at the anterior pituitary to selectively suppress FSH synthesis and secretion, although their relative biological potencies differ [66]. In addition to its direct pituitary action, inhibin may also influence hypothalamic GnRH regulation indirectly through antagonism of activin signaling, as activin has been reported to modulate GnRH neuronal activity and GnRH secretion in hypothalamic systems [67,68,69]; however, the relative importance of this hypothalamic pathway compared with the direct pituitary action of inhibin remains less well established [67]. Through this negative-feedback regulation of FSH, inhibin influences follicular recruitment and subsequent follicular development and maturation [18]. FSH also directly supports granulosa-cell steroidogenic function, including estradiol production, as demonstrated in cultured bovine granulosa cells [70,71]. Because reducing inhibin bioactivity can increase circulating FSH, inhibin has been investigated as an endocrine target to promote follicular development and ovulation responses in livestock species [72,73]. Experimental studies using inhibin immunization approaches have demonstrated increased FSH secretion, enhanced follicular development, and improved ovulation responses in ruminants, including buffaloes and sheep [72,73]. Beyond its canonical role in suppressing pituitary FSH secretion, inhibin-related signaling also contributes to local ovarian cellular functions [74,75,76]. Inhibin A promotes granulosa-cell proliferation and suppresses apoptosis in porcine and bovine granulosa cells [74,75]. Consistently, in Hu sheep granulosa cells, *INHBA* knockdown inhibited cell-cycle progression, promoted apoptosis, and reduced inhibin A secretion [76].

In addition to endocrine regulation by inhibin, intra-ovarian TGF-β superfamily members, particularly BMP15 and GDF9, modulate granulosa-cell proliferation and FSH-dependent steroidogenic activity [20,77,78]. These oocyte-derived factors fine-tune follicular growth, maturation, and ovulatory competence and therefore act in concert with inhibin-mediated regulation of FSH to coordinate follicle selection and ovulatory success [20,77,78]. Another TGF-β superfamily member relevant to ovarian follicular dynamics is anti-Müllerian hormone (AMH). AMH, secreted by granulosa cells in females (and Sertoli cells in males), limits excessive early follicle recruitment and serves as a marker of ovarian reserve [62,79]. Studies have shown that variations in AMH levels are associated with ovarian activity, ovulation-related traits, and litter size in small ruminants, with supporting evidence in sheep populations [80,81,82].

The reproductive response to inhibin suppression varies with both the neutralization strategy and the strength of the resulting immunological response. This conclusion is based on studies using inhibin vaccines, in which the immunogen targeted the common inhibin α-subunit rather than inhibin A or inhibin B specifically; the vaccine constructs incorporated the porcine inhibin α(1–32) sequence fused to hepatitis B surface antigen S (HBsAg-S) [72,73]. Alternative strategies have involved passive immunization approaches, including anti-inhibin serum and anti-inhibin monoclonal antibodies generated against a 27-amino-acid peptide of the porcine inhibin α-subunit [11]. However, these studies generally assessed antibody production and associated reproductive outcomes, including follicular development, ovulation, conception, and litter size, rather than directly determining the extent of circulating inhibin reduction [11,72,73]. Consequently, because the interventions and outcome measures are not directly comparable, clear numerical thresholds defining “moderate,” “intermediate,” or “severe” inhibin suppression cannot currently be established. Accordingly, the available evidence is discussed in terms of the neutralization approach and the magnitude of the associated reproductive response.

Several studies have indicated that immunoneutralization of inhibin can enhance follicular development and reproductive performance without apparent adverse effects on pregnancy outcome [11,72,73]. In crossbred buffaloes, vaccination against the common inhibin α-subunit, specifically the α(1–32) sequence, induced anti-inhibin antibodies and increased the number and diameter of large follicles; the highest vaccine dose increased the ovulation rate from 1.02 ± 0.12 in controls to 1.49 ± 0.19 [72]. Similarly, inhibin α-subunit DNA vaccination increased the number of mature follicles and litter size in rats and increased the twinning rate in sheep from 10% in controls to 39.2% in immunized animals [73]. More recently, anti-inhibin monoclonal antibody treatment, using an antibody generated against a 27-amino-acid peptide antigen of the porcine inhibin α-subunit, increased the number of surviving offspring from 8.6 to 11.9 pups in C57BL/6 mice and from 15.3 to 21.2 pups in ICR mice [11]. These findings are consistent with the established role of inhibin as a negative regulator of FSH and demonstrate that controlled neutralization can enhance ovarian responsiveness and reproductive output [39,40].

Increasing the ovarian response following inhibin neutralization beyond an optimal range does not necessarily result in a proportional increase in viable offspring. Hasegawa et al. [11] showed that anti-inhibin serum induced a greater ovulatory response than treatment with the monoclonal antibody (raised against a 27-amino-acid peptide of the porcine inhibin α-subunit and targeting the common α-subunit shared by both inhibin A and inhibin B). Although anti-inhibin serum treatment increased the number of implantation sites, this increase was accompanied by greater post-implantation losses and did not result in a corresponding increase in surviving pups [11]. In contrast, the less pronounced ovarian response to monoclonal-antibody treatment (24.6 normal oocytes per female compared with 51.8 following anti-inhibin serum treatment) was associated with significantly more surviving offspring [11]. A similar biological limitation has been observed in rabbits selected for 10 generations based on ovulation rate, a physiological reproductive trait rather than circulating inhibin or another biochemical parameter: approximately two additional ova were obtained after selection, but litter size did not improve because prenatal survival declined [85]. Although the rabbit study did not target inhibin levels directly, it supports the broader principle that increasing ovulation beyond the reproductive capacity of the female may not improve viable litter size.

### 2.4. Mechanistic Basis of Inhibin Action: Antagonism of Activin Signaling

**Figure 4:**
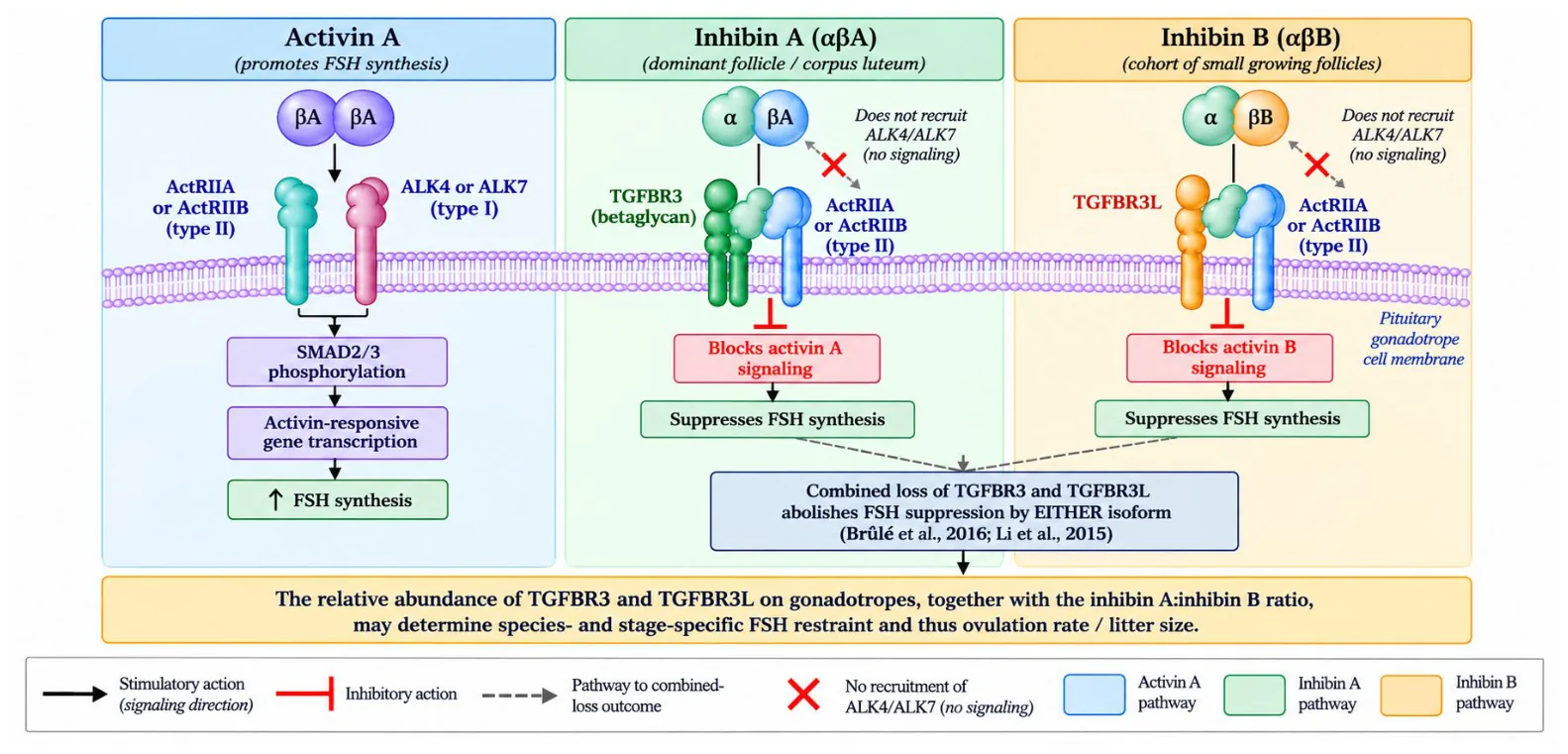
Mechanistic basis of inhibin-mediated antagonism of activin signaling and isoform-specific co-receptor usage. Activin A binds activin type II receptors (ActRIIA/ActRIIB) and recruits the type I receptors ALK4/ALK7, leading to SMAD2/3 phosphorylation and activin-responsive transcription that promotes FSH synthesis. In contrast, inhibin A and inhibin B bind activin type II receptors through their β-subunits and use distinct co-receptors, with TGFBR3 (betaglycan) supporting inhibin A and TGFBR3L preferentially supporting inhibin B. Inhibin binding does not recruit ALK4/ALK7; instead, the resulting receptor complexes antagonize activin signaling and suppress FSH synthesis. Combined loss of TGFBR3 and TGFBR3L abolishes FSH suppression by either inhibin isoform [87,88]. The relative abundance of TGFBR3 and TGFBR3L, together with the inhibin A: inhibin B ratio, is proposed as a potential determinant of stage- and species-specific FSH regulation and, consequently, ovulation rate and litter size. The comparative relationship between co-receptor abundance and reproductive output remains a working hypothesis requiring validation in livestock. The figure was prepared by the authors based on the experimental findings reported in [87,88], and the receptor-interaction evidence summarized in Table 1.

Mechanistically, inhibin does not initiate intracellular signaling independently; instead, it primarily antagonizes activin-dependent signaling that would otherwise promote FSH synthesis [40]. Activin typically signals by binding type II receptors (ActRIIA/ActRIIB) and recruiting a type I receptor (ALK4/ALK7), activating downstream phosphorylation events that culminate in SMAD2/3 nuclear translocation and transcription of activin-responsive genes relevant to pituitary function [40]. In contrast, inhibin antagonizes activin signaling through a distinct receptor mechanism. The β-subunits of inhibin interact with activin type II receptors, while accessory co-receptors such as betaglycan (TGFBR3) for inhibin A and TGFBR3L for inhibin B facilitate high-affinity binding and prevent productive assembly of the activin receptor complex required for downstream SMAD2/3 activation [86,87,88]. Thus, inhibin suppresses activin signaling not by initiating an alternative signaling pathway, but by limiting activin receptor complex formation and reducing activin-mediated transcriptional responses [86,87,88,89,90]. The receptor mechanisms underlying inhibin-mediated antagonism of activin signaling, including the isoform-specific involvement of TGFBR3 and TGFBR3L, are summarized in Figure 4.

In inhibin A, the α-subunit-containing complex promotes high-affinity antagonism through recruitment of the co-receptor betaglycan (TGFBR3) [86,89,90]. For inhibin B, the related co-receptor TGFBR3L contributes to effective antagonism [87,88]. These interactions generate stable, non-signaling receptor complexes that restrict activin receptor assembly and downstream signaling [86,87,88,89,90]. The specific residues forming the betaglycan-binding surface of the α-subunit and the type II receptor-binding interface of the β-subunit are therefore key molecular determinants of inhibin-mediated activin antagonism; however, residue-level characterization of these interaction surfaces has been derived predominantly from human and rodent experimental systems [39,86,87,88].

**Table 1:** Structural and functional evidence defining inhibin interactions with betaglycan and activin type II receptors.

Molecular Interface	Key Residues/Region or Binding Property	Experimental Approach	Principal Finding	Evidence Interpretation	References
Inhibin A α-subunit–betaglycan	Tyr^50^, Val^108^, Thr^111^, Ser^112^, Phe^118^, Lys^119^, Tyr^120^; additional contributions from Arg^109^, Thr^110^ and Ser^117^	Site-directed mutagenesis, radioligand competition binding and homology modelling	These residues form a contiguous betaglycan-binding epitope on the outer convex surface of the mature α-subunit	Direct residue-level evidence for the inhibin A–betaglycan interface	[91]
Inhibin A α-subunit–betaglycan	Val^108^–Tyr^120^	Mutagenesis and binding assays	V108L reduced betaglycan affinity ~6-fold; K119A/Y120A reduced affinity ~25-fold	Major region determining high-affinity betaglycan binding	[91]
Inhibin A α-subunit–betaglycan	T111A/S112A/Y120A	Mutagenesis, purified-protein binding and pituitary-cell bioassays	Strongly impaired betaglycan binding and inhibin A FSH-suppressive activity; WT IC_50_ = 85 pM in LβT2 cells, with >70-fold loss of potency in the mutant, and WT IC_50_ = 25 pM in primary rat pituitary cells, with ≥40-fold loss of potency.	Demonstrates functional requirement for high-affinity betaglycan binding	[91]
βA-subunit–ActRIIB	Phe^17^, Ile^30^, Ala^31^, Pro^32^, His^36^, Arg^87^, Pro^88^, Ser^90^, Leu^92^, Tyr^94^, Ile^100^, Lys^102^, Glu^111^	X-ray crystallography of activin A–ActRIIB complex	ActRIIB contacts the outer convex/knuckle surface of βA through hydrophobic and polar/ionic interactions	Direct structural evidence for activin A; relevant to the shared βA-subunit of inhibin A	[92]
βA-subunit–ActRIIB	Ile^30^, Ser^90^, Leu^92^, Ile^100^	Site-directed mutagenesis, receptor-binding and functional signaling assays	Mutations at these positions impaired ActRIIB binding and activin biological activity	Direct functional evidence defining important βA receptor-binding determinants	[93]
ActRIIB–βA-subunit	Tyr^60^, Trp^78^, Phe^101^; additional contacts involving Val^73^, Leu^79^, Phe^82^ and Val^99^	X-ray crystallography	Central aromatic cluster forms the hydrophobic core of the ActRIIB–βA interface	Direct receptor-side structural evidence	[92]
ActRII–activin/inhibin	Phe^42^, Trp^60^, Phe^83^	Alanine-scanning mutagenesis, radioligand binding and reporter assays	F42A, W60A and F83A abolished detectable activin A and inhibin A binding	Direct receptor-side functional evidence	[94]
Inhibin A–betaglycan–ActRII	No individual residues mapped	Binding, cross-linking and functional cell assays	Betaglycan binds inhibin A with high affinity, enhances its association with ActRII, and confers cellular responsiveness to inhibin A	Establishes betaglycan/TGFBR3 as a functional co-receptor for inhibin A	[86]
Inhibin A/B–betaglycan	Inhibin A IC_50_ ≈329 pM; inhibin B IC_50_ ≈533 pM	Competition binding	Both inhibin isoforms bind betaglycan with similarly high affinity	Supports interaction through the common α-subunit; no residue mapping performed	[95]
Inhibin A/B–ActRIIB2	Relative affinity: activin A > inhibin B > inhibin A	Competition binding and cross-linking	Both inhibins bind ActRIIB2; inhibin B binds more strongly than inhibin A	Demonstrates β-subunit-dependent isoform differences	[95]
Inhibin A/B–ActRIIB2–betaglycan	No individual residues mapped	Receptor co-expression and competition binding	Betaglycan increased inhibin A affinity for ActRIIB2, but did not significantly increase inhibin B affinity	Isoform-dependent co-receptor effect	[95]
Inhibin A/B–ActRIIA–betaglycan	No individual residues mapped	Receptor co-expression and binding assays	Betaglycan significantly enhanced binding of both inhibin A and B to ActRIIA	Betaglycan particularly facilitates inhibin interaction with ActRIIA	[95]

Abbreviations: ActRIIA—activin receptor type IIA; ActRIIB—activin receptor type IIB; TGFBR3—transforming growth factor beta receptor type III (betaglycan); IC_50_—half-maximal inhibitory concentration. Note: Residue numbers for inhibin subunits refer to positions within the mature subunit sequences; residue numbers for receptor proteins refer to the corresponding mature receptor sequences.

As summarized in Table 1, residue-level studies have identified a betaglycan-binding epitope on the outer convex surface of the inhibin α-subunit involving Tyr^50^, Val^108^, Thr^111^, Ser^112^, Phe^118^, Lys^119^, and Tyr^120^, with the Val^108^–Tyr^120^ region particularly important for inhibin A activity [91]. Because both inhibin A and inhibin B contain a β-subunit (βA in inhibin A, βB in inhibin B), structural and mutational studies of the βA-subunit (which is shared between inhibin A and activin A) have indicated that Ile^30^, Ser^90^, Leu^92^, and Ile^100^ are important determinants of its interaction with ActRIIB [92,93]. On the receptor side, Phe^42^, Trp^60^, and Phe^83^ of ActRII are required for both activin A and inhibin A binding [94].

As a high-affinity inhibin co-receptor, betaglycan enhances the interaction of inhibin with activin type II receptors [86]. However, this effect is isoform- and receptor-dependent: inhibin A and B bind betaglycan with similarly high affinity, but betaglycan preferentially enhances inhibin A binding to ActRIIB2 while facilitating binding of both isoforms to ActRIIA [95].

Importantly, co-receptor-mediated antagonism is not equivalent for the two inhibin isoforms, and this asymmetry may be central to interspecies variation in reproductive output rather than a purely descriptive detail. Gonadotrope betaglycan (TGFBR3) functions preferentially as a co-receptor for inhibin A [87], whereas the more recently characterized TGFBR3L is selective for inhibin B [88]; the two co-receptors are differentially required for effective inhibin-mediated suppression of FSH, and combined loss of both abolishes suppression by either isoform [87,88]. As summarized schematically in Figure 4, these isoform-specific co-receptor interactions provide a mechanistic basis for differential inhibin A- and inhibin B-mediated regulation of FSH. Because inhibin A and inhibin B have distinct, stage-dependent secretion patterns [96], and because TGFBR3 and TGFBR3L show isoform-specific roles in inhibin-mediated FSH suppression [87,88], differences in the relative contribution of these co-receptors could influence which inhibin isoform predominates in regulating FSH at different reproductive stages. This provides a potential mechanistic framework for investigating whether species- and tissue-specific differences in co-receptor expression, in addition to differences in inhibin secretion, contribute to variation in ovulation rate and litter size; however, this proposed relationship requires direct validation in livestock [87,88].

Experimental studies provide evidence that altering inhibin activity can modify FSH secretion and ovulation rate in ruminants, while manipulation of inhibin co-receptor pathways produces corresponding reproductive effects in mice [87,88]. In ewes, immunoneutralization of inhibin increased circulating FSH and ovulation rate, demonstrating that reduced inhibin-mediated negative feedback can promote follicular recruitment [97]. In mouse models, loss of TGFBR3L increased FSH levels, ovarian follicle development, and litter size, whereas combined loss of TGFBR3L and betaglycan resulted in infertility [88]. Together, these findings suggest that the magnitude of inhibin activity and the receptor mechanisms through which inhibin A and inhibin B suppress FSH may influence reproductive output. However, whether differences in the relative expression of TGFBR3 and TGFBR3L, or in the inhibin A: inhibin B ratio, explain species-specific differences in ovulation rate and litter size remains untested. Comparative assessment of TGFBR3 and TGFBR3L expression, together with inhibin A and inhibin B concentrations, across ruminant and non-ruminant livestock could therefore provide a direct test of this proposed mechanism.

## 3. Role of Inhibin in Litter Size of Ruminants and Non-Ruminants

The role of inhibin in litter size regulation is rooted in the FSH-suppressing negative feedback, as described in Section 2. Table 2 and Table 3 summarize the reported variants in *INHA*, *INHBA*, *INHBB*, and related regulatory factors associated with litter size or other reproductive traits in ruminants. Collectively, these studies identify several potentially informative markers; however, the strength of evidence varies substantially among breeds and populations. Importantly, for many reported associations, effects on inhibin expression, circulating inhibin concentrations, FSH secretion, or protein bioactivity have not been experimentally demonstrated. Therefore, these variants should generally be interpreted as candidate markers of prolificacy rather than confirmed causal determinants. The equivalent data for non-ruminant species are summarized in Section 3.2.

### 3.1. Inhibin’s Role in Regulating Litter Size in Ruminants

Several independent studies have reported associations between *INHA* polymorphisms and litter size in small ruminants [98,99,100]. These associations are frequently breed- or population-specific. In Boer goats, the heterozygous 651A/G genotype was associated with approximately 0.27 more offspring per litter than the corresponding homozygous genotypes [98]. In a study of one prolific and five low- to medium-prolificacy Chinese goat breeds, two *INHA* polymorphic regions were identified. At this locus, referred to as the P4 locus, a G→A transition at the 759th position of the amplified fragment, corresponding to G841A of the *INHA* gene, was detected across all six breeds. In Jining Grey goats, Bb does produced, on average, 0.79 more kids than BB does (*P* < 0.01), whereas genotype-specific litter-size associations were not evaluated in the other five breeds [101]. The reported *INHA* variants associated with litter size in ruminants are summarized in Table 2.

In Hainan Black goats, 211 does were genotyped, and litter-size records were available for 90 individuals. Among seven identified *INHA* SNPs, the exon 2 missense variant g.28317663A>C was significantly associated with litter size; does carrying the AC genotype showed a higher mean litter size (1.583 ± 0.504) than AA genotype carriers (1.349 ± 0.480; *P* = 0.046) [102]. The variant was predicted to cause a Thr-to-Pro substitution; however, whether this amino acid change affects inhibin secretion, receptor interaction, or biological activity has not been experimentally validated.

In Dazu Black goats, 190 does with 534 litter records were analyzed to evaluate associations between reproductive traits and genetic variants. The study identified one *INHA* variant (chr2:g.28314990T>G) and two *ACVR2B* variants significantly associated with litter size (*P* < 0.05) [103]. For the *INHA* variant, heterozygous and alternate homozygous animals showed estimated increases of 0.12 and 0.25 kids per litter, respectively, compared with the reference homozygous genotype [103]. Although these findings suggest that *INHA* variation may contribute to litter-size differences, functional experiments are still required to determine whether these variants directly influence inhibin production or reproductive physiology.

Additional evidence comes from West African Dwarf, Red Sokoto, and Kalahari Red goats. Isa et al. [104] examined 226 West African Dwarf, 70 Red Sokoto, and 70 Kalahari Red goats and identified four *INHA* SNPs. At the 3′UTR g.3234C>T locus, West African Dwarf does with the CT genotype had significantly larger litter sizes than those with the CC genotype (*P* = 0.01). The significant litter-size association was confined to the West African Dwarf population, while the remaining identified loci were not significantly associated with litter size [104].

Additional *INHA* associations with litter size have been reported in sheep and several goat populations [100,101,105]. In Indonesian Thin-Tailed sheep, the synonymous exon 2 g.236311367G>A (p.Pro300Pro) variant was associated with litter size, although the study included only 45 ewes and the GA and AA genotypes were represented by only two and one animals, respectively [101]. In Malabari goats (*n* = 251), the exon 2 c.911T>C missense variant (Val299Ala) was associated with litter size; PP (*n* = 199) and PQ (*n* = 41) does had litter sizes of 1.59 ± 0.04 and 1.57 ± 0.05, respectively, compared with 1.42 ± 0.05 in QQ does (*n* = 11; *P* ≤ 0.05) [100]. Hou et al. [99]., further reported that *INHA* P1 promoter-region genotypes were associated with litter size in Xinong Saanen, Guanzhong, and Boer goats, with the AA genotype showing greater litter size (*P* < 0.05). Collectively, these studies support associations between *INHA* variation and prolificacy, but the evidence does not establish a common causal mechanism. Some associated variants are synonymous or non-coding and may exert regulatory effects or be in linkage.

Variation in the β-subunit genes has also been associated with reproductive traits. In Hu sheep, the *INHBB* 276A>G mutation was associated with litter size, with the BB genotype having approximately 0.58 more lambs than the AA genotype [106]. 

As summarized in Table 3, polymorphisms in *INHBA* have also been linked to prolificacy in Egyptian sheep and goats, where the P1 genotype was associated with higher twinning rates, whereas P2 and P3 were associated with lower litter size [107]. In Xu et al.’s multi-breed GWAS, the top litter size-associated SNP in Finnsheep (rs412280524) fell within a linkage-disequilibrium block containing *INHBB*, whereas the top signal in Icelandic sheep from the same study (rs429836421) mapped to *NCOA1* rather than an inhibin-subunit gene. Only the Finnsheep INHBB-linked signal is therefore treated here as an inhibin-pathway association [108]. Overall, the evidence summarized in Table 2 and Table 3 supports associations between variation in inhibin-related genes and reproductive performance in several ruminant populations. Nevertheless, most studies remain association-based, and relatively few have directly linked a specific genotype to altered inhibin synthesis, secretion, bioactivity, or downstream FSH regulation.

**Table 2:** Inhibin α-subunit (*INHA*) variants associated with litter size in ruminants.

Species (Breed)	Gene/Variant	Location/Region	Coding Consequence & Predicted Effect on Protein	Reproductive Trait Assessed	Key Findings	References
Goat (Jining Grey + five Chinese indigenous breeds)	*INHA* P4 locus, reported as G759A/G841A	Exon 1	Synonymous substitution; AAG → AAA (Lys → Lys), with no amino-acid change. Any phenotypic effect would therefore most likely arise through a regulatory mechanism or linkage disequilibrium with another functional allele	Litter-size association assessed in Jining Grey goats; P4 genotype frequencies determined in six goat breeds	BB and Bb genotypes were detected in all six breeds. In Jining Grey goats, Bb does (*n* = 261) produced 2.80 ± 0.09 kids compared with 2.01 ± 0.19 for BB does (*n* = 24; *P* < 0.01). Litter-size associations were not evaluated in the other five breeds	[101]
Sheep (Indonesian Thin-Tailed)	*INHA* g.236311367G>A	Exon 2	Synonymous; functional basis not reported	Litter size	GA ewes had significantly higher litter size than AA or GG ewes (*P* < 0.05)	[105]
Goat (Boer)	*INHA* 651A/G	Exon 2	Not reported; overdominance implies heterozygote advantage	Litter size (2nd parity)	Heterozygotes ~0.27 more kids than homozygotes; overdominance suggested	[98]
Goat (West African Dwarf, Red Sokoto, and Kalahari Red)	*INHA* g.3234C>T (rs670889338)	3′-UTR	May influence post-transcriptional regulation or be in linkage disequilibrium with a causal variant; functional effect not experimentally validated	Litter size	WAD (*n* = 226), RS (*n* = 70), and KR (*n* = 70). In WAD goats, the CT genotype was associated with significantly larger litter size than CC (*P* = 0.01); no significant litter-size associations were reported for the other loci	[104]
Goat (Hainan Black)	*INHA* g.28317663A>C	Exon 2	Missense; predicted Thr316Pro substitution; structural consequences predicted in silico but not functionally validated	Litter size	AC (*n* = 24): 1.583 ± 0.504 vs. AA (*n* = 66): 1.349 ± 0.480; *P* = 0.046; 90 does with litter-size records	[102]
Goat (Dazu Black)	*INHA* chr2:g.28314990T>G (SNP_1)	Exon 2	Nonsynonymous substitution; functional effect on inhibin bioactivity not experimentally demonstrated	Litter size (1st–3rd parity)	In 190 does (534 litter records), the variant showed significant additive and dominant associations; heterozygous and alternate-homozygous genotypes had estimated effects of +0.12 and +0.25 kids relative to the reference genotype	[99]
Goat (Xinong Saanen, Guanzhong, Boer)	*INHA* P1 locus (PCR-RFLP)	5′ promoter region	Functional effect not directly demonstrated	Litter size	AA, AB, and BB genotypes were detected in all three breeds. The AA genotype was associated with greater litter size than the other genotypes at the P1 locus (*P* < 0.05).	[99]
Goat (Malabari)	*INHA* c.129G>A (exon 1); c.911T>C (exon 2)	Exons 1 and 2	Not reported	Litter size	Exon 1: AA lower than AB; exon 2: PP and PQ higher than QQ	[100]
Sheep (Tianmu polytocous)	*INHA*-associated A/T SNP	Chr 2: 221477155; upstream of *INHA*	Functional consequence not experimentally demonstrated	Litter size (1st parity)	T/A had greater litter size than T/T (*P* < 0.05), and A/A had greater litter size than T/T (*P* < 0.01); no significant difference between T/A and A/A was indicated.	[109]

Note: For variants marked “Not reported,” we checked the Ensembl and NCBI dbSNP databases where an rs identifier or an unambiguous genomic coordinate was available in the primary study. This allowed the region to be confirmed for some entries (e.g., Table 3); where the primary study reported only a position within a PCR amplicon or SSCP fragment, without a deposited rs identifier or the primer sequences needed to re-map the fragment to the reference genome, the subgenic location could not be independently confirmed and is retained here as “Not reported” rather than inferred.

**Table 3:** *INHBA*, *INHBB*, and related regulatory factors associated with reproductive traits in ruminants.

Species (Breed)	Gene/Variant	Location/Region	Coding Consequence & Predicted Effect on Protein	Reproductive Trait Assessed	Key Finding	References
Sheep (Hu)	*miR-134-3p* targeting *INHBA*	3′-UTR	Lowers *INHBA* mRNA and intracellular protein in cultured granulosa cells (in vitro reporter/transfection assay; not measured as circulating inhibin A)	Granulosa-cell proliferation/apoptosis; follicular development	*miR-134-3p* targets *INHBA*, inhibits proliferation and promotes apoptosis via TGF-β/PI3K/AKT/mTOR	[110]
Sheep (Finnsheep)	*INHBB (rs412280524*, LD block)	LD block containing INHBB; intragenic position not confirmed	Not reported; opposite direction of effect between breeds	Litter size	A/A genotype had significantly higher litter size than A/G genotype (2.84 vs. 2.08 lambs, *P* < 0.001)	[108]
Goat (Xinong Saanen, Guanzhong, Boer)	*INHA* P1 locus: C80G and −/126G (single-nucleotide G insertion/deletion at position 126; numbering according to GenBank HQ202573)	5′ promoter region	Functional effect not demonstrated	Litter size	AA, AB, and BB genotypes were detected at the P1 locus in all three breeds. The AA genotype was associated with greater litter size in Xinong Saanen, Guanzhong, and Boer goats (*P* < 0.05). A total of 786 goats from the three breeds were examined.	[99]
Sheep (Hu)	*INHBB* 276A>G	3′-UTR	Functional effect not experimentally demonstrated	Litter size (prolificacy)	AA, AB and BB genotype frequencies were 0.636, 0.046 and 0.318, respectively; BB ewes produced 0.58 more lambs than AA ewes (*P* < 0.01)	[106]
Cattle (Iraqi Holstein Friesian)	Inhibin A/Activin A β-subunit SNPs [A(877)R, C(1865)Y, C(883)S]	Not reported	Nomenclature implies amino-acid substitutions; structural effect not reported	Superovulation, FSH and inhibin A during estrus	SNPs more frequent in high-twinning (DZTB) cows; lower inhibin A, higher FSH; C(883)S and A(877)R affected ovulation and calving	[111]
Sheep (Egyptian breeds)	*INHBA* polymorphisms detected with primers 1, 3 and 5	Exon 1 (primer 1) and exon 2 (primers 3 and 5); ovine *INHBA* mapped to chromosome 4q26	Sequence variants identified; amino-acid consequences were not established in this study	Litter size/twin production	95 sheep were genotyped; 83 mothers were used for litter-size association. At the exon-1 primer-1 locus, AA mothers (*n* = 59) had litter size 1.74 ± 0.10, CC (*n* = 21) 1.47 ± 0.24, and AC (*n* = 3) 1.00 ± 0.49. At the exon-2 primer-3 locus, AA mothers (*n* = 63) had greater litter size than GG mothers (*n* = 20): 1.75 ± 0.19 vs. 1.20 ± 0.70 (*P* < 0.05)	[107]
Goat (Egyptian breeds)	*INHBA* polymorphism detected with primer 4	Exon 2 (primer 4; amplified region 728–1060)	Sequence variants identified; amino-acid consequences were not established	Litter size/twin production	44 goats were genotyped; 30 mothers were used for association. GG mothers (*n* = 22), AA (*n* = 6), and AG (*n* = 2) had litter sizes of 2.09 ± 0.28, 2.17 ± 0.18, and 2.50 ± 0.50, respectively; the AG group showed the highest litter size, although its sample size was only two mothers.	[107]

Note. “Location” gives the position of the variant within the gene where reported. “Coding consequence” states whether the variant alters the mature protein (non-synonymous), leaves it unchanged (synonymous), acts post-transcriptionally, or was not characterized in the source study. “Not reported” means the primary study did not establish the variant’s position relative to the coding sequence or its effect on protein structure, stability, or activity, so that the mechanistic basis of the association remains unvalidate.

#### 3.1.1. Indirect Regulators of Inhibin-Related Reproductive Traits

In contrast to variants within the inhibin-subunit genes, several genetic and molecular pathways may influence inhibin-related reproductive processes indirectly through effects on granulosa-cell function, follicular development, and intra-ovarian signaling [110,112,113,114,115,116,117,118,119]. The FecL mutation involving *B4GALNT2* provides a distinct indirect mechanism of inhibin modulation. Rather than altering the sequence of an inhibin-subunit gene, the mutation causes ectopic expression of *B4GALNT2* in ovarian granulosa cells, which has been associated with altered glycosylation and reduced inhibin A concentration or bioactivity [29,112,113]. Thus, this pathway may modify the post-translational properties and biological activity of inhibin without directly changing the *INHA* sequence.

Post-transcriptional regulation represents another indirect mechanism. In Hu sheep granulosa cells, miR-134-3p has been reported to target *INHBA*, thereby affecting granulosa-cell proliferation and apoptosis and influencing cellular responses associated with inhibin A [110]. In this case, regulation occurs at the RNA level rather than through a sequence variant within the inhibin gene itself. Similarly, mutations in BMP15, GDF9, and *BMPR1B* affect intra-ovarian signaling, follicular development, and oocyte–granulosa-cell communication [115,116,117,118,119]. These genes therefore represent upstream or parallel components of the follicular regulatory network rather than direct modifiers of the inhibin molecule. Any alteration in inhibin expression or activity associated with these pathways may consequently arise secondary to changes in follicular development or granulosa-cell function.

Collectively, these examples illustrate that genetic and molecular regulation of inhibin-related reproductive traits can occur through pathways outside the inhibin-subunit genes themselves. Such indirect mechanisms may influence inhibin abundance or activity through altered glycosylation, post-transcriptional regulation, follicular development, or granulosa-cell function. However, the extent to which these pathways alter inhibin bioactivity and downstream FSH regulation remains insufficiently characterized in livestock. Functional studies integrating gene expression, inhibin A/B abundance or bioactivity, FSH concentrations, and follicular phenotypes will therefore be required to establish the mechanistic contribution of these indirect regulators.

#### 3.1.2. Potential and Limitations of Inhibin-Related Markers for Marker-Assisted Selection in Ruminants

Marker-assisted selection has been proposed as an approach for improving litter size and reproductive efficiency in small ruminants [120,121]. However, the variants summarized in Table 2 and Table 3 differ substantially in the strength of supporting evidence. Candidate markers are more compelling when an association is reproduced across independent breeds or populations, supported by a strong statistical signal and adequate genotype numbers, or involves a coding change with a plausible effect on the mature inhibin protein.

As summarized in Table 2, several loci warrant particular attention for further validation. The *INHA* P1 promoter-region polymorphism was associated with litter size in Xinong Saanen, Guanzhong, and Boer goats, providing evidence of association across three goat breeds [99]. The *INHBB*-linked signal in Finnsheep showed one of the strongest statistical associations, with A/A animals having a mean litter size of 2.84 compared with 2.08 in A/G animals (*P* < 0.001) [108], although the causal variant within the associated region remains unresolved. In Jining Grey goats, the synonymous *INHA* P4-locus variant reported as G759A/G841A also showed a relatively large phenotypic difference, with Bb does producing 2.80 ± 0.09 kids compared with 2.01 ± 0.19 in BB does (*P* < 0.01) [101]. Similarly, the *INHBB* 276A>G association in Hu sheep was associated with approximately 0.58 additional lambs in BB compared with AA animals (*P* < 0.01) [106]. These associations are statistically notable, but independent replication remains limited.

**Figure 5:**
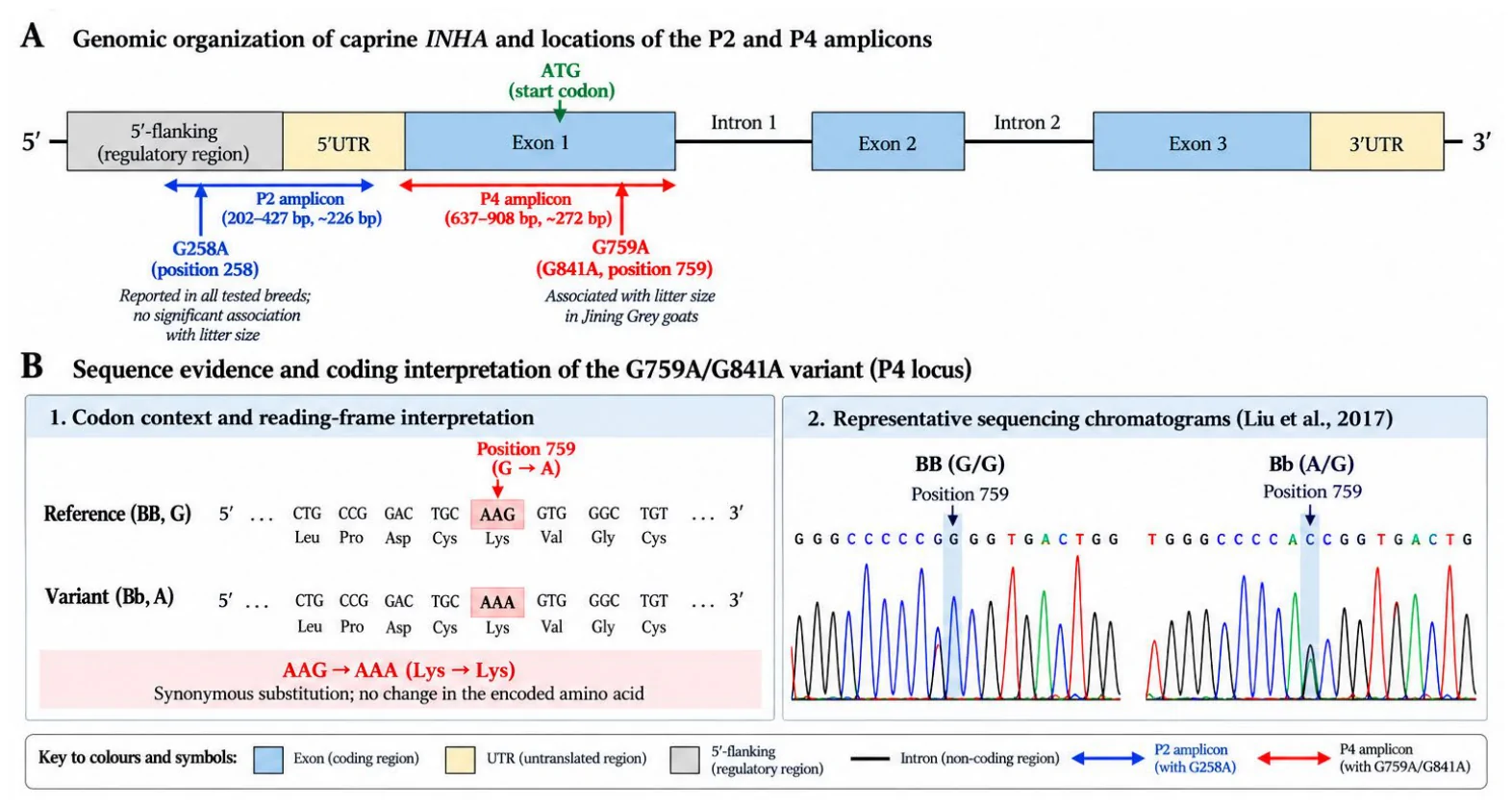
Genomic context and coding-sequence interpretation of caprine *INHA* variants reported by Liu et al. (2017) [101]. (**A**) Schematic organization of the caprine *INHA* gene showing the 5′-flanking region, 5′UTR, exons, introns, 3′UTR, and the regions amplified by primers P2 and P4. The G258A variant was identified in the P2 amplicon, whereas the G759A/G841A variant was identified in the P4 amplicon and was associated with litter size in Jining Grey goats. (**B**) Sequence context and coding interpretation of the G759A/G841A variant. The G→A substitution changes the AAG codon to AAA, both encoding lysine (Lys), and therefore represents a synonymous coding substitution. Representative sequencing chromatograms for the BB (G/G) and Bb (A/G) genotypes are shown. The figure was prepared as a schematic representation based on the findings reported by Liu et al. [101], to clarify the genomic context and sequence interpretation of the reported variants; it is not a reproduction of the original sequencing figure.

The Jining Grey *INHA* G759A/G841A finding also illustrates the importance of distinguishing the presence of a polymorphism from replication of its phenotypic association. The variant was detected across six goat breeds, but the association with litter size was reported only in Jining Grey goats [101]. The variant is located downstream of the ATG start codon within exon 1 and changes AAG to AAA, both of which encode lysine; thus, it represents a synonymous coding variant rather than a 5′-UTR variant. The genomic location and reading-frame interpretation are illustrated in Figure 5. Although a synonymous substitution does not alter the amino-acid sequence, its association could potentially reflect effects on mRNA stability, splicing, translation efficiency, or linkage disequilibrium with another functional variant. However, these possibilities remain untested for this specific variant and should not be interpreted as demonstrated mechanisms.

Missense variants provide an additional category of biologically plausible candidates. In Hainan Black goats, *INHA* g.28317663A>C was predicted to produce a Thr316Pro substitution and was associated with litter size (AC: 1.583 ± 0.504 vs. AA: 1.349 ± 0.480; *P* = 0.046) [102]. In Malabari goats, the exon 2 c.911T>C variant produces a Val299Ala substitution; PP and PQ animals had larger litter sizes than QQ animals (1.59 ± 0.04 and 1.57 ± 0.05 vs. 1.42 ± 0.05; *P* ≤ 0.05) [100]. Both residue positions lie within the C-terminal region corresponding to the mature inhibin-α subunit in mammalian inhibin processing, making these variants particularly relevant for functional investigation, although neither has yet been shown experimentally to alter inhibin secretion or bioactivity. Mammalian inhibin-α precursors are processed to generate the mature C-terminal α-domain, with cleavage occurring around residues 227–233 in bovine and human proteins.

Other associations require greater caution. The g.3234C>T association in West African Dwarf goats was significant (*P* = 0.01), but comparable evidence was not obtained in Red Sokoto or Kalahari Red goats, where the minor allele was uncommon [104]. Likewise, the Indonesian Thin-Tailed sheep association for the synonymous g.236311367G>A variant was based on only 45 animals, including two GA and one AA ewe, despite the reported *P* < 0.05 [105]. Such findings should therefore be considered preliminary until reproduced in larger populations with adequate representation of the less frequent genotypes.

Taken together, the evidence summarized in Table 2 and Table 3 indicates that the Finnsheep *INHBB*-linked signal, the Jining Grey *INHA* G759A/G841A association, and the Hu sheep *INHBB* 276A>G variant are notable on the basis of statistical significance and/or effect size, whereas the *INHA* g.28317663A>C and c.911T>C variants are particularly interesting because they are predicted to alter the mature inhibin-α subunit. However, these criteria identify priority candidates rather than establish causality. No variant currently combines convincing independent replication, strong statistical support, and direct experimental evidence of altered inhibin function.

Accordingly, future studies should prioritize independent validation in sufficiently large populations with adequate numbers of each genotype and appropriate consideration of breed, parity, management, and environmental effects. Studies should report allele frequencies, genotype-specific effect sizes, confidence intervals, and relevant covariates to facilitate comparison among populations. Fine-mapping and haplotype analyses are particularly important for synonymous and regulatory variants to determine whether the reported marker itself is functional or is linked to another causal locus.

Functional characterization should receive particular attention for missense variants affecting the mature inhibin-α region, including Thr316Pro and Val299Ala. Comparative studies of wild-type and variant proteins could determine whether these substitutions affect precursor processing, mature protein formation, secretion, dimer assembly, receptor/co-receptor interactions, or inhibition of activin signaling. For synonymous and non-coding variants, reporter assays, allele-specific expression, transcript analysis, and assessment of mRNA stability or processing could help establish their molecular consequences.

A further priority is to connect genotype directly with endocrine and reproductive phenotypes within the same animals. Concurrent measurement of circulating inhibin A and B, FSH, follicular recruitment, follicle number and size, ovulation rate, embryo survival, and final litter size would help determine whether an associated variant acts through the inhibin–FSH axis or merely tracks prolificacy through linkage with another locus. Such integrated genotype–endocrine–phenotype studies would also help determine whether increased ovulation translates into increased numbers of viable offspring.

Finally, future validation should consider reproductive efficiency beyond litter size alone, including conception rate, embryo and fetal survival, offspring survival, and relevant maternal traits. This is important because selection for increased ovulation or litter size may not necessarily result in a proportional improvement in viable offspring production. Thus, the currently reported inhibin-related variants should be regarded as priority candidates for further validation rather than universally established markers for routine marker-assisted selection in ruminants.

### 3.2. Inhibin’s Role in Regulating Litter Size in Non-Ruminants

In non-ruminants, inhibin is produced mainly by granulosa cells of developing follicles and functions as a selective negative-feedback regulator of circulating FSH [122,123,124]—the mechanism of which is described in full in Section 2—thereby modulating the cohort of growing follicles [122,123], and ovulation rate—two proximal determinants of litter size when prenatal survival is adequate [122,123,124,125,126]. Experimental evidence further supports this endocrine role: partial neutralization of inhibin (and, in some models, co-targeting inhibin with other ovarian regulators such as AMH) elevates circulating FSH, increases the cohort of growing follicles, and can improve ovarian steroidogenic output [55,56] and fecundity outcomes, including litter size, depending on the extent of embryonic and fetal survival [11]. Experimental modulation of inhibin activity has provided additional functional evidence. Active immunization of gilts against a recombinant bovine inhibin α-subunit increased mean ovulation rate by approximately 35%, with ovulatory response positively associated with serum inhibin-binding activity [127].

#### 3.2.1. Inhibin and Litter Size in Pigs

Among non-ruminant livestock, pigs provide an important model for evaluating the relationship between inhibin-mediated follicular regulation and genetic variation in prolificacy because litter size is a highly selected and economically important reproductive trait in commercial pig production [128,129]. In pigs, inhibin contributes to ovarian follicular development through regulation of the hypothalamic–pituitary–gonadal axis; however, evidence linking inhibin-pathway variation with naturally occurring differences in litter size remains limited.

Molecular studies in pigs provide evidence supporting the involvement of inhibin signaling in reproductive regulation. Kempisty et al. [128], demonstrated that *INHA* and *INHB* transcripts and proteins are expressed in porcine oocytes and follicular cells, with expression patterns differing among follicles of different developmental stages. These findings indicate that inhibin signaling participates in follicular differentiation, oocyte maturation, and ovarian function. Nevertheless, physiological involvement of a gene does not necessarily indicate that genetic variation within that gene represents a major determinant of population-level differences in reproductive traits.

At the candidate-gene level, polymorphisms within the inhibin pathway have been examined in pigs. In 189 Suhuai pigs, a −42G>A polymorphism in the 5′-UTR of the *INHA* gene was significantly associated with litter size, with GG animals producing 0.63 more piglets per litter than AA animals (*P* < 0.05) [130]. Because the identified polymorphism was located in a regulatory region, it was proposed that genetic variation within *INHA* might influence reproductive performance through altered gene expression or inhibin production. Nevertheless, direct functional evidence demonstrating effects on *INHA* transcription, circulating inhibin concentration, FSH secretion, follicular recruitment, or ovulation rate was not provided. Therefore, this association should currently be considered a potential genetic marker rather than a confirmed causal mutation.

Experimental studies have also examined whether inhibin neutralization alters reproductive outcomes in pigs. Passive immunization of 173 Yorkshire sows against an inhibin α-subunit peptide attenuated the normal postweaning decline in FSH in a dose-dependent manner but did not significantly affect farrowing rate or total or live litter size [131]. In a separate study, pre-weaning immunization against a recombinant porcine inhibin α-subunit increased live litter size compared with untreated controls (10.79 ± 0.23 vs. 9.96 ± 0.37 piglets; *P* = 0.044), whereas the increase in total litter size showed only a trend toward significance (10.99 ± 0.24 vs. 10.22 ± 0.38; *P* = 0.068) [132]. Collectively, these findings indicate that experimental reduction in inhibin bioactivity can alter FSH regulation and selected reproductive outcomes in pigs, although increased FSH secretion does not invariably translate into increased final litter size.

Recent genome-wide association studies (GWASs) have provided a broader perspective on the genetic architecture of litter size in pigs and allow evaluation of whether inhibin-pathway genes represent major contributors to prolificacy. Cielen et al. [133] conducted a GWAS using whole-genome sequence data from 40,509 Large White sows, including 27,035,402 SNPs, to identify genomic regions associated with total number born (TNB) and litter-size variability. The study identified a significant quantitative trait locus (QTL) on *Sus scrofa* chromosome 1 (~164.8 Mb), with *SMAD3*, *SMAD6*, and *MAP2K1* highlighted as candidate genes associated with mean litter size [133]. These genes are involved in TGF-β signaling, embryonic development, and reproductive regulation. Importantly, no significant associations were detected for the classical inhibin subunit genes *INHA*, *INHBA*, or *INHBB*, suggesting that although inhibin signaling contributes to ovarian physiology, genetic variation within these genes may have limited effects on overall litter-size variation.

Similarly, Chen et al. [134] investigated population genomic differences associated with reproductive performance using whole-genome resequencing data from six pig populations with different litter-size characteristics, including high-prolific Large White and Jishen Black pigs. By integrating fixation index (F_ST), nucleotide diversity ratio (π-ratio), and cross-population extended haplotype homozygosity (XP-EHH), the authors identified 12 putative candidate genes associated with litter-size differentiation, including *KCNQ1*, *PEX14*, *CDK15*, *SPAG17*, *TTF2*, *CD101*, *CASQ2*, *VANGL1*, *ARID5B*, *KLHL32*, *EML1*, and *NAV1* [134]. Among these candidates, *KCNQ1* showed consistent selection signals and ovarian expression, indicating a possible role in follicular development and reproductive performance. However, similar to the GWAS results of Cielen et al. [133], inhibin-related genes were not identified as major targets of selection. The available evidence regarding the relationship between inhibin-pathway genes and porcine litter-size variation is summarized in Table 4.

#### 3.2.2. AMH as a Fertility Marker in Companion Animals

In addition to inhibin-related markers, AMH—closely linked to follicle dynamics—has also been explored as a fertility marker in veterinary medicine, including mares and dogs [135,136,137,138,139,140,141,142,143,144,145]. In bitches, AMH has shown associations with litter size in some studies (e.g., an approximate increase of 0.3 puppies per 1 ng/mL AMH in one report). Still, findings are not fully consistent across cohorts and may be confounded by factors such as body weight and sampling time during the reproductive cycle, indicating the need for larger, well-controlled datasets and harmonized reference intervals before routine predictive use [146,147]. In dogs, serum AMH concentrations vary throughout the estrous cycle, increasing from late anestrus and decreasing during the final days before ovulation [148]. In domestic cats, serum AMH concentration, together with age, has been proposed as an indirect predictor of ovarian reserve [149]. Table 5 summarizes reported inhibin- and AMH-related reproductive markers in non-ruminant species, highlighting their proposed roles in ovarian function, fertility assessment, and litter-size variation.

**Table 4:** Summary of genetic and genomic evidence linking inhibin-related pathways with litter-size variation in pigs.

Species/Breed	Gene/Genomic Region	Experimental Approach	Main Findings	Biological Interpretation	References
Pig (crossbred Landrace gilts)	*INHA*, *INHB*	Expression analysis of porcine oocytes isolated from small (<3 mm), medium (3–5 mm), and large (>5 mm) follicles before and after in vitro maturation	*INHA* and *INHB* transcripts and proteins showed differential expression and cellular distribution according to follicular size and IVM status. *INHA* mRNA was increased in oocytes from large follicles, whereas *INHB* mRNA increased progressively with follicular size after IVM. Differential protein localization of both inhibins was also observed	Provides evidence for differential inhibin expression in porcine oocytes during follicular development and oocyte maturation; however, the study does not establish a direct contribution to litter-size variation	[128]
Suhuai pig	*INHA* 5′-UTR variant	Candidate-gene polymorphism association analysis	A regulatory-region polymorphism within *INHA* was associated with reproductive traits	Suggests a possible regulatory effect of *INHA* variation, but functional effects on inhibin production, FSH regulation, or litter size require validation	[130]
Large White pig	SSC1 (~164.8 Mb); *SMAD3*, *SMAD6*, *MAP2K1*	Whole-genome sequence GWAS (40,509 sows; 27,035,402 SNPs)	Significant QTL associated with mean total number born (TNB) identified on SSC1; candidate genes included *SMAD3*, *SMAD6*, and *MAP2K1*	Indicates that TGF-β signaling and developmental pathways may contribute to variation in litter size	[133]
Large White pig	SSC13 region near *TFF3* and *UMODL1*	Whole-genome sequence GWAS for litter-size variability	Rare variants associated with variation in litter-size stability among sows	Suggests genomic regulation of reproductive robustness and phenotypic stability	[133]
Multiple pig populations (Large White, Jishen Black, Berkshire, etc.)	*KCNQ1*, *PEX14*, *CDK15*, *SPAG17*, and other candidate genes	Whole-genome resequencing; F_ST, π-ratio, and XP-EHH selection-signature analyses	Twelve putative candidate genes associated with differences among populations with divergent litter-size performance; *KCNQ1* showed consistent selection signals and ovarian expression	Suggests that multiple reproductive pathways, rather than inhibin genes alone, contribute to population differences in prolificacy	[134]
Pig populations	*INHA*, *INHBA*, *INHBB*	Genome-wide association and selection-signature analyses	No genome-wide significant associations or major selection signals were detected for classical inhibin subunit genes	Indicates that inhibin has an established physiological role but currently limited detectable contribution to genetic variation in porcine litter size	[133,134]

**Table 5:** Reported associations of inhibin- and AMH-related reproductive markers with fertility and litter-size traits in non-ruminant species.

Species	Inhibin/AMH Function	Effect on Ovulation Rate	Effect on Litter Size/Fertility	Key Findings	References
Bitches	*AMH* secreted by granulosa cells; marker of ovarian reserve	No direct effect found	Higher *AMH* correlates with larger litters	An increase of approximately 0.3 puppies per litter was reported for each 1 ng/mL increase in serum AMH	[146]
Bitches	*AMH* as fertility marker during proestrus/estrus	No direct effect indicated	Higher *AMH* linked to larger litters	Serum AMH measured during proestrus and estrus was associated with litter size; the relationship was influenced by body weight	[147]
Bitches	Serum AMH as an indicator of ovarian activity throughout the estrous cycle	No direct effect on ovulation rate	Litter size was not evaluated	Serum AMH concentrations changed throughout the estrous cycle, increasing from late anestrus and decreasing before ovulation	[148]
Queens/domestic cats	Serum AMH as an indirect predictor of ovarian reserve	No direct effect on ovulation rate was assessed	Ovarian reserve was evaluated; litter size was not assessed	Serum AMH was positively associated with primordial follicle number, while age was inversely associated; AMH and age predicted ovarian reserve	[149]
Queens/domestic cats	Serum AMH as an indirect marker of ovarian follicle size and functionality	Ovulation rate was not assessed	Ovarian follicle population was assessed; litter size was not evaluated	Serum AMH decreased with age and was strongly correlated with small antral follicle number (r = 0.85, *P* < 0.01), supporting its use as an indirect marker of ovarian follicle size and functionality	[150]

#### 3.2.3. Inhibin and Litter-Size Regulation in Rabbits

In rabbits, litter size is determined principally by ovulation rate and prenatal survival [124]. Selection experiments have demonstrated that increasing ovulation rate alone does not necessarily increase litter size because gains in the number of ova can be offset by reduced prenatal survival [85]. This provides an important physiological context for interpreting inhibin-mediated increases in follicular recruitment and ovulation.

Direct evidence linking naturally occurring *INHA* variants with litter-size differences in rabbit populations remains limited. The rabbit *INHA* gene is annotated on chromosome 7 and represents a biologically plausible candidate for fertility-related studies, but robust genotype–phenotype associations between inhibin-pathway variants and recorded litter-size traits have not yet been established [151]. Consequently, current evidence for a role of *INHA* in rabbit prolificacy is largely inferential and based on the established physiological role of inhibin in regulating FSH and ovulation rather than on direct population-level genetic evidence.

This represents an important knowledge gap. Future studies should combine sequencing of *INHA* and other inhibin-pathway genes with measurements of circulating inhibin A/B, FSH, ovulation rate, prenatal survival, and final litter size in the same animals. Such integrated analyses would clarify whether inhibin-related genetic variation contributes meaningfully to prolificacy in commercial rabbit populations.

#### 3.2.4. Selection Trade-Offs and Welfare Considerations

Finally, selection focused on a single trait can yield unfavorable correlated responses—particularly when higher ovulation is accompanied by increased prenatal loss and reduced offspring viability—so integrative breeding decisions should consider multiple components (mean litter size, variability, survival, and maternal capacity) rather than litter size alone [152]. Selection for alleles that moderately reduce inhibin bioactivity should be accompanied by monitoring of prenatal survival and offspring viability to ensure that any increase in ovulation rate translates into viable litter-size gains rather than simply elevated embryonic loss. Even practical questions such as whether replacement gilts should be selected from small versus large litters remain unresolved, reflecting competing influences of early-life environment and inherited prolificacy [153,154,155]; this uncertainty reinforces a broader gap in translating inhibin-related endocrine or genetic findings into consistent, welfare-aligned improvements under field conditions [156,157].

#### 3.2.5. Selection Trade-Offs and Knowledge Gaps in Non-Ruminants

Increasing ovulation rate does not necessarily produce a proportional increase in viable litter size because reproductive output also depends on fertilization, prenatal survival, and maternal capacity. Experimental evidence in mice demonstrates this trade-off: anti-inhibin monoclonal antibody treatment increased the number of surviving offspring, whereas stronger ovarian stimulation with anti-inhibin serum produced a greater ovulatory response but did not yield a corresponding improvement in surviving offspring because post-implantation losses increased [11]. Similarly, selection for increased ovulation rate in rabbits produced approximately two additional ova but did not increase litter size because prenatal survival declined [85]. These findings indicate that the reproductive benefit of enhancing ovarian response should be evaluated using viable offspring rather than ovulation rate alone.

Comparable trade-offs are relevant to pig breeding. Selection for increased prolificacy has increased litter size, but very large litters are associated with greater risks of low birth weight, mortality, and welfare problems [156,157]. Maternal capacity, including the ability to support offspring before and after birth, can therefore limit the realized benefit of increasing litter size. Consequently, selection strategies should consider prenatal survival, offspring viability, litter-size variability, and maternal capacity together rather than focusing exclusively on the number of ovulations or offspring born [152,156,157,158,159].

Important knowledge gaps also remain in translating inhibin-related findings into practical selection strategies. In pigs, reported associations between *INHA* variation and reproductive performance require further functional validation to determine whether the associated variants alter *INHA* expression, circulating inhibin, FSH secretion, or ovarian response [130]. In rabbits, direct population-level evidence linking naturally occurring *INHA* or other inhibin-pathway variants with ovulation rate and litter size remains limited [151]. More broadly, candidate inhibin-related markers should be validated across populations, parities, and management conditions, with simultaneous assessment of genotype, inhibin A and B concentrations, FSH, ovulation rate, prenatal survival, and viable litter size. Such integrated studies are required to determine whether modulation of the inhibin pathway can provide consistent reproductive benefits without compromising offspring survival or animal welfare [85,156,157].

## 4. Verification of Inhibin Function in Rodent Models

Mouse and rat studies provide direct functional evidence that inhibin regulates fecundity primarily through pituitary control of FSH [160,161,162], but that the reproductive outcome depends on the degree of inhibin attenuation [11,163]. Across models, partial reduction in inhibin bioactivity (or its pituitary actions) tends to elevate FSH-driven follicle recruitment, increase ovulation rate, and enlarge litter size, whereas more severe disruption of inhibin signaling can impair follicular function and reduce overall reproductive output, including subfertility despite elevated FSH [11,163]. Consistent with this endocrine logic, FSH synthesis in pituitary gonadotropes is stimulated by hypothalamic GnRH and activin-class ligands, while gonadal inhibins suppress FSH and act as functional antagonists of activin-class proteins in this regulatory circuit [86,88,160,161,162,164].

### 4.1. Genetic Models Targeting Inhibin Co-Receptors

Mechanistic evidence for the role of inhibin co-receptors in FSH suppression and fertility has been strengthened by genetic models targeting inhibin co-receptors on gonadotropes [87,88]. Betaglycan (TGFBR3) binds inhibin A and inhibin B, whereas TGFBR3L shows inhibin B selectivity, and both are required for effective inhibin-mediated suppression of FSH in mice [87,88]. Gonadotrope-specific betaglycan (TGFBR3) knockout females and global *Tgfbr3l* knockouts display increased litter sizes [87,88], accompanied by enhanced folliculogenesis and ovulation; in *Tgfbr3l* knockout mice, these changes were associated with increased FSH, whereas FSH levels were not detectably altered in the gonadotrope-specific *Tgfbr3* knockout model [87,88]. Inhibin A fails to suppress FSH in cultured pituitaries lacking gonadotrope betaglycan [88], and combined loss of betaglycan and TGFBR3L abolishes suppression by both inhibin A and inhibin B [87]. However, models producing inhibins with markedly reduced bioactivity show that elevated FSH does not guarantee higher fertility, because excessive attenuation can compromise ovarian or gestational competence and lead to subfertility [162,163].

### 4.2. Interventional Studies and Immunization Approaches

Interventional studies align with the genetic evidence that reducing inhibin signaling can alter FSH-dependent follicular development and reproductive output. In female mice, co-immunization with plasmids encoding *INHA* and *AMH* increased estradiol levels and litter size (reported ~15.45 pups, approximately 1.6 times higher than controls) without affecting offspring birth weight [165], suggesting that combined modulation of inhibin and AMH signaling can influence reproductive output. Lower *DAZL* expression in oocytes enhances granulosa-cell responsiveness to FSH [166], enabling more follicles to mature and ovulate under reduced FSH, which increases corpora lutea and ultimately increases litter size in mice. Complementary experimental evidence comes from the use of anti-inhibin serum (AIS), which has been used to raise endogenous FSH and establish superovulation protocols effective across multiple laboratory and wild-derived mouse strains [167,168,169,170]. For benchmarking reproductive output, Swiss Webster ICR mice are frequently used because of their high fecundity and typical litter sizes of ~10–15 pups [11,171]. Complementary rodent evidence also indicates that increasing inhibin tone can be detrimental: overexpression of the inhibin α-subunit in rats was associated with impaired reproductive performance, including a litter size that was approximately 52% lower and longer intervals between pregnancies [172,173]. These findings collectively demonstrate that optimal reproductive outcomes require balanced inhibin signaling—neither too high nor too low. Table 6 summarizes representative rodent-model studies investigating how experimental manipulation of inhibin signaling influences reproductive outcomes.

## 5. Conclusions

Inhibin plays a fundamental role in reproductive regulation by controlling pituitary FSH secretion, thereby influencing follicular recruitment, development, maturation, and ovulation rate, which are key physiological determinants of litter size. Evidence from functional studies and genetic analyses indicates that inhibin-mediated regulation of folliculogenesis contributes to reproductive variation across livestock species; however, the magnitude of its contribution differs among species and populations. Genetic studies have identified associations between inhibin-related genes, particularly *INHA*, and prolificacy-related traits in several ruminant populations, whereas evidence in non-ruminants remains comparatively limited, with recent genome-wide studies in pigs suggesting that litter-size variation is predominantly influenced by multiple genomic regions involved in follicular development, embryonic survival, and maternal reproductive capacity rather than by inhibin-subunit genes alone. Functional studies further demonstrate that partial modulation of inhibin activity can enhance FSH secretion, follicular recruitment, ovulation rate, and litter size under appropriate physiological conditions, whereas excessive disruption of inhibin signaling may impair reproductive efficiency. Therefore, inhibin should be considered as an important endocrine regulator of reproductive processes, while its role as a major genetic determinant of litter-size variation requires further clarification.

Despite substantial progress, several challenges remain before inhibin-related information can be effectively applied in livestock breeding programs. Many reported associations between inhibin-pathway variants and reproductive traits are derived from candidate-gene studies, and direct functional validation of causal variants remains insufficient. In addition, species-specific differences in ovarian physiology, follicular dynamics, and litter-size regulation limit the direct extrapolation of findings among livestock species. Future research should integrate genome-wide association studies, transcriptomics, epigenomics, proteomics, and endocrine profiling to establish stronger links between inhibin-related genetic variation, hormone regulation, follicular characteristics, ovulation rate, embryo survival, and final litter size. Such integrative approaches will help to determine whether inhibin-pathway variants can serve as reliable reproductive markers and support balanced breeding strategies that improve prolificacy while maintaining offspring viability, maternal performance, and animal welfare.

## Figures and Tables

**Table 6:** Inhibin pathway perturbations and reproductive performance in rodent models.

Species	Inhibin Function	Effect on Ovulation Rate	Effect on Litter Size/Fertility	Key Findings	References
C57BL/6J Mice	Anti-inhibin monoclonal antibody (AIMA)	3.5-fold increase	~3.5-fold increase	AIMAs significantly increased oocytes, implantation, and surviving pups	[11]
Mice	INHA + AMH co-immunization	Attenuates FSH-induced steroidogenesis	15.45 pups (1.6× control)	Higher estradiol; no effect on birth weight	[165]
Mouse (Dazl het)	Elevated inhibin B marks accelerated follicle maturation	Increased (more CL)	Increased litter size	Follicles more FSH-sensitive; more ovulations	[166]
Mouse (transgenic α-subunit overexpression)	Excessive FSH suppression	~54% fewer oocytes	~52% reduction	Excessive inhibin impairs female fecundity	[172]
Rats	Anti-inhibin mAb superovulation	~1.3× in Wistar	~1.4–1.5× litter size	Enhanced efficiency vs. eCG/hCG	[174]

## Data Availability

No new data were created or analyzed in this study. Data sharing is not applicable to this article.
